# Nutritional status and function after high-calorie formula vs. Chinese food intervention in undernourished children with cerebral palsy

**DOI:** 10.3389/fnut.2022.960763

**Published:** 2022-10-06

**Authors:** Yiting Zhao, Lu He, Tingting Peng, Liru Liu, Hongyu Zhou, Yunxian Xu, Xubo Yang, Yuan Huang, Zhaofang Chen, Yi Xu, Jinling Li, Xiaohui Hou, Hongmei Tang, Kaishou Xu

**Affiliations:** ^1^Department of Rehabilitation, Guangzhou Women and Children's Medical Center, Guangzhou Medical University, Guangzhou, China; ^2^Department of Sports and Health, Guangzhou Sport University, Guangzhou, China; ^3^School of Medicine, South China University of Technology, Guangzhou, China

**Keywords:** undernutrition, cerebral palsy, high-calorie formula, z-score, Chinese food

## Abstract

**Aim:**

To investigate the efficacy and safety of high-calorie formula vs. Chinese daily food on the nutritional status and motor function of undernourished children with cerebral palsy (CP).

**Methods:**

In this prospective, assessor-blind, and randomized controlled trial, we recruited children (1–10 years) with CP and undernutrition based on the WHO and the American Society for Parenteral and Enteral Nutrition criteria from the National Children's Medical Center. Participants were randomly allocated (1:1) to a high-calorie formula group or a Chinese daily food diet group (control group) for 6 months. Indirect calorimetry was used to estimate energy requirements. We compared the nutritional status and gross motor function of participants in both groups based on weight, height, *z*-scores (weight-for-age, height-for-age, weight-for-height, and BMI-for-age), and the Gross Motor Function Measure (GMFM), respectively, at baseline, 3-, and 6-months follow-up. In addition, the effective rate of nutritional intervention, and adverse events were simultaneously assessed.

**Results:**

From July 2020 to December 2021, a total of 119 participants were enrolled and randomized, and 110 participants completed the study (with 54 children in the high-calorie formula group and 56 children in the control group). After 6 months of treatment, the weight, height, z-scores (weight-for-height, weight-for-age, and BMI-for-age), and GMFM of both groups were significantly improved (*p* < 0.05). There were significant differences in changes in weight, weight-for-age z-scores, and GMFM between the two groups (*p* < 0.05). During the study period, 16 children experienced at least one mild adverse event [9 (16.7%) in the formula group and 7 (12.5%) in the control group].

**Conclusion:**

Nutritional intervention with a high-calorie formula may be an effective and safe option in children with CP for improving undernutrition and gross motor dysfunction.

**Clinical trial registration:**

www.chictr.org.cn, identifier: ChiCTR2000033878.

## Introduction

Cerebral palsy (CP) is an early-onset, non-progressive neuromotor disorder of the brain in children with multiple neurological deficits and a complex molecular etiology (1). In addition to motor impairment, undernutrition is a prominent comorbidity in children with CP. According to published studies, about 22.2–76.6% of children with CP are undernourished (2, 3). Children with CP often suffer from dysphagia, feeding difficulty, gastroesophageal reflux, slow gastric emptying, and digestion, which usually result in undernutrition and growth failure (4). There are many reasons for undernutrition in children with CP, e.g., diarrhea, increased muscle tone, and the presence of involuntary movements. One of the main reasons is inadequate intake because of eating and drinking dysfunction (5). Except for the failure to thrive, decreased muscle strength, an impaired immune system, and an increased risk of infection may occur in undernourished children. Furthermore, cognitive and motor development may be affected when children are combined with chronic undernutrition (6). Therefore, it is essential that undernutrition in this group of children is recognized promptly and treated appropriately to prevent secondary bad outcomes.

Enteral nutrition is the preferred way to meet children's energy and nutrient requirements (7). A randomized controlled trial showed that feeding the protein and energy-enriched formula promoted more adequate nutrient intake and facilitated energy and nitrogen balance in infants with a critical illness (8). Another retrospective study suggested that a high-calorie formula (1.0 kcal/ml) could help the infants in the intensive care unit gain weight (9). However, there are few studies and limited data on the nutritional intervention of children with CP currently. For neurologically impaired children, clinical guidelines recommended a high-calorie formula as the preferred way to provide enteral nutrition support (10). It is recommended to feed a neurologically impaired child with enteral formulas containing multi-nutrients that suffice for long-term use (11), but daily food diets are still prevalent and widely used in China because most Chinese families believe that such food is more natural and healthier. On the other hand, the types and components of the daily diet in different countries may vary owing to different food cultures and culinary habits. Moreover, the effectiveness of nutritional intervention between high-calorie formulas and Chinese daily food diets in this population remains unclear.

The prospective, randomized, controlled clinical trial, therefore, aimed to investigate the efficacy of high-calorie formula and Chinese daily food diets on nutritional status and gross motor function of undernourished children with CP.

## Methods

### Participants

In total, 213 children were assessed for eligibility, of which 145 met eligibility criteria and 119 gave consent and were randomized. Finally, 110 participants completed the study (with 54 children in the high-calorie formula group). Recruitment occurred from July 2020 to December 2021. Inclusion criteria were as follows: (i) children who were diagnosed as CP (12); (ii) children aged 1–10 years; (iii) children who were combined with undernutrition; (iv) children on oral feeding [the Eating and Drinking Ability Classification System (EDACS) or Mini-EDACS levels I–III]; and (v) children who had not received nutritional intervention within 6 months. Participants were excluded if they met any of these criteria: (i) other neurological diagnoses; (ii) uncontrolled seizures; (iii) cow's milk protein allergy; (iv) severe gastrointestinal diseases; and (v) parenteral nutrition.

### Study design

This study was a prospective, randomized controlled, and assessor-blind trial, which was part of a larger study and was registered at chictr.org.cn (ChiCTR2000033878). This study was approved by the Research Ethics Committee of Guangzhou Women and Children's Medical Center (Approval Number: 202023301), and written informed consent was obtained from the legal guardian of each participant before enrollment.

Participants were assigned to either a high-calorie formula or a Chinese diet group in a 1:1 ratio randomly using a random number table produced by the R Programming Language (version 3.6.1). Assessments and administration of the functional scales and questionnaires were performed by the two independent qualified assessors. Assessors were blind to group assignment. Children were evaluated at baseline, 3, and 6 months after randomization.

### Interventions

The weight and height of the included children were measured. Stevenson's equation [height (cm) = (tibial length × 3.26) + 30.8] (13) was used to estimate the height of participants whose actual height was unavailable due to joint contracture and deformity. Nutritional status was defined by the weight-for-age *z*-score (WAZ), height-for-age *z*-score (HAZ), weight-for-height *z*-score (WHZ), and body mass index (BMI)-for-age *z*-score (BAZ), which were calculated *via* the WHO Anthro (version 3.2.2, for children <5 years) or AnthroPlus software (version 1.0.4, for children ≥ 5 years). Then, nutritional status was categorized into mild (−2 < WHZ or BAZ ≤ −1), moderate (−3 < WAZ, HAZ, WHZ, or BAZ ≤ −2), or severe undernutrition (WAZ, HAZ, WHZ, or BAZ ≤ −3) according to the WHO growth charts (14) combined with the American Society for Parenteral and Enteral Nutrition (ASPEN) standards (15).

Indirect calorimetry (10) was used to estimate energy requirements: energy intake (kcal/day) = basal metabolic rate × muscle tone × activity + growth, where: muscle tone = 0.9 if decreased, 1.0 if normal, and 1.1 if increased; activity = 1.1 if bedridden, 1.2 if wheelchair dependent or crawling, and 1.3 if ambulatory; and growth = 5 kcal/g of desired weight gain (normal and catch-up growth). The basal metabolic rate was estimated by the Schofield equation (16). Participants in the formula group were administrated with a high energy density polymeric formula (1.0 kcal/ml, Nutren Junior, Nestle; or Peptamen Junior, Nestle) for 6 months. Specifically, subjects with normal gastrointestinal function were fed with 50% whey and 50% casein whole-protein formula (Nutren Junior), otherwise, subjects received the 100% whey partially hydrolyzed protein formula (Peptamen Junior), which could help to digest and absorb. Participants in the control group were solely fed with a Chinese diet for nutritional supplementation during the study period. Parents received personal nutritional counseling and feeding instruction from the clinical dietitian.

Additionally, both the formula and control groups received an individual physical therapy program based on a goal-directed, task-focused approach and motor learning theory (17). The types of activities provided in the study included strength training, over-ground training, and goal-directed training, which were tailored to the specific condition of the included participants. The frequency (5 times a week), intensity (three sets of 8–12 repetitions before fatigue), and time (30 min per session, for 24 consecutive weeks) (18) of exercise training were identical in both groups. Hospital-centered exercise was carried out in the hospital for the first 10 sessions, and family-centered training was subsequently conducted until the end of the study. Telephone and Wechat follow-up and rehabilitation guidance were performed every 2 weeks.

### Outcome measures

Assessments were performed at the baseline visit, 3, and 6 months post-intervention. The gross motor function and eating and drinking ability of the participants were identified at baseline. Gross motor functions were classified by the Gross Motor Function Classification System (GMFCS), which is a wellestablished tool to assess the severity of motor impairment in children with CP (19). Eating and drinking abilities were determined by the EDACS or Mini-EDACS, which are the standard levels for swallowing function (20, 21).

The z-scores, weight, and height were assessed to identify the nutritional status. Changes in the gross motor function were measured by the Gross Motor Function Measure (GMFM), which is a validated tool to quantify the gross motor function and has excellent reliability and validity in children with CP (22). The GMFM has been widely assessed for the effectiveness of interventions in children with CP (23, 24). After 3 and 6 months of intervention, the number of children whose nutritional status had changed to normal in each group was recorded and compared. Adverse events, mainly gastrointestinal intolerance, were evaluated through the parent-reporting of symptoms, updating medical records, and physician review. Gastrointestinal intolerance related to the nutritional intervention was assessed during the whole study period.

### Statistical analysis

Data analyses were performed with SPSS version 26.0 (IBM Corp., Armonk, NY, USA). For continuous variables, student's *t*-test or the Wilcoxon rank-sum test was performed to compare the baseline data between the two groups. For categorical variables, the chi-square test or the Fisher exact test was analyzed. Repeated measures analysis of variance (ANOVA) and simple effect analysis were performed for the within-group differences of WAZ, HAZ, WHZ, BAZ, weight, height, and GMFM data. The Mann–Whitney *U*-test was used for comparisons of changes between the independent group mean ranks. Level information was expressed by frequency and percentage. Statistical significance was set at *p* < 0.05.

## Results

### Demographic and clinical characteristics

**Figure 1:**
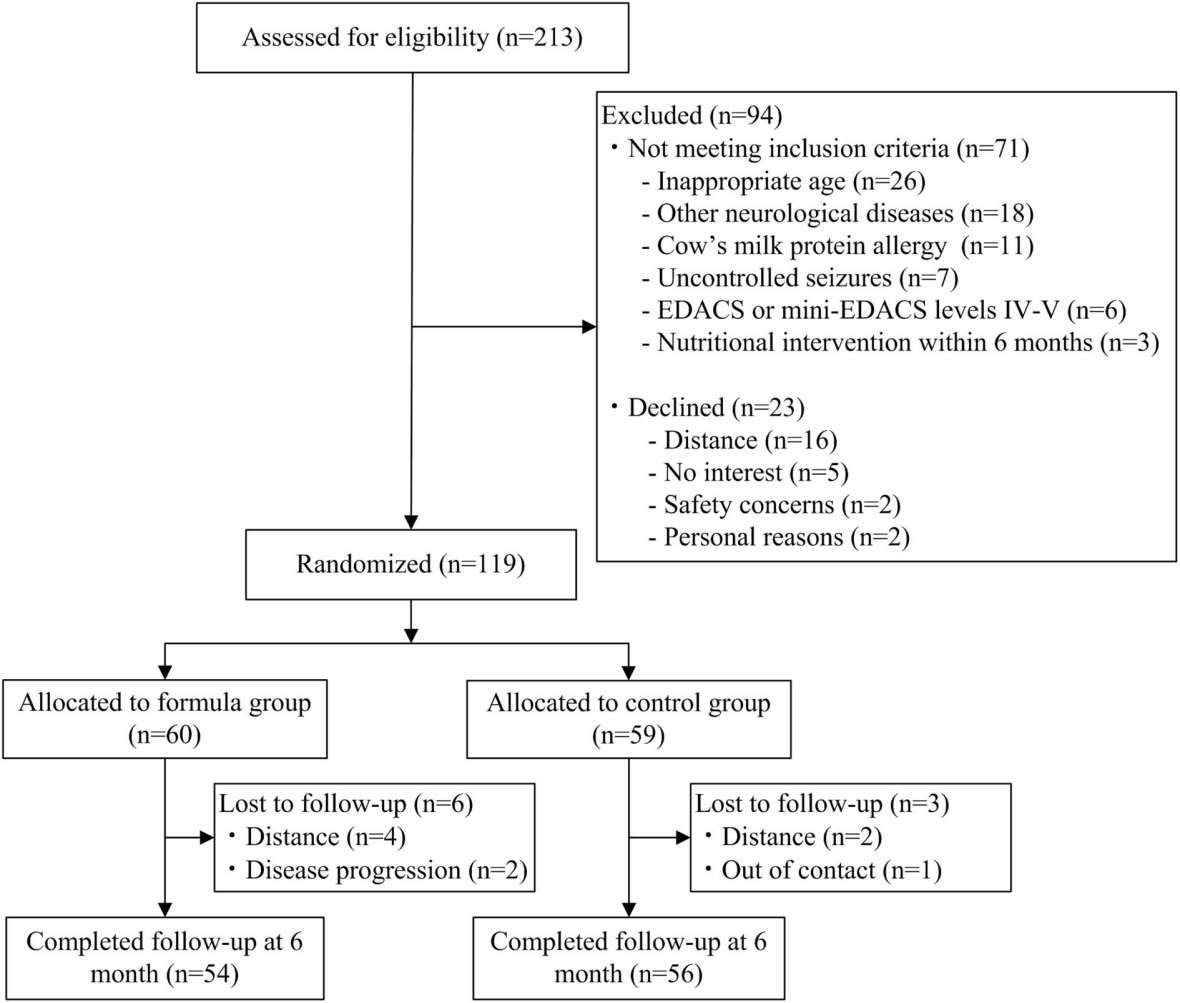
Consolidated standards of reporting trials flowchart.

A total of 110 participants (74 males and 36 females) completed the study. Spastic cerebral palsy (*n* = 105, 95.5%) was the most common type. Most of the children (*n* = 82, 74.5%) were in GMFCS levels I–III, while 25.5% (*n* = 28) were in GMFCS levels IV and V. There were no significant differences in baseline demographic characteristics, functional performance, and nutritional parameters between the two groups (Table 1). Participant flow is shown in Figure 1.

**Table 1:** Demographic and clinical characteristics.

	**Formula group** **(*n* = 54)**	**Control group** **(*n* = 56)**	***p-*value**
Gender, male/female	33/21	41/15	0.176
Age (m)	35.8 ± 22.9	35.6 ± 18.7	0.428
CP type			0.806
Spastic	51 (94.4)	54 (96.4)	
Dyskinetic	2 (3.7)	2 (3.6)	
Mixed	1 (1.9)	0 (0)	
GMFCS level			0.808
I	17 (31.5)	15 (26.8)	
II	10 (18.5)	12 (21.4)	
III	14 (25.9)	14 (25.0)	
IV	6 (11.1)	10 (17.9)	
V	7 (13.0)	5 (8.9)	
EDACS/Mini-EDACS level			0.341
I	36 (66.7)	42 (75.0)	
II	8 (14.8)	9 (16.1)	
III	10 (18.5)	5 (8.9)	
Undernutrition classification			0.521
Mild	24 (44.4)	19 (33.9)	
Moderate	24 (44.4)	29 (51.8)	
Severe	6 (11.1)	8 (14.3)	
Weight (kg)	11.16 ± 3.06	11.28 ± 2.71	0.620
Height (cm)	87.98 ± 12.54	88.29 ± 11.38	0.654
Weight-for-height z-score	−1.35 ± 0.70	−1.35 ± 1.05	0.652
Weight-for-age z-score	−1.82 ± 0.77	−1.92 ± 0.96	0.486
Height-for-age z-score	−1.61 ± 1.16	−1.78 ± 1.33	0.466
Body mass index-for-age z-score	−1.20 ± 0.74	−1.19 ± 1.09	0.511
Gross Motor Function Measure	53.76 ± 32.08	54.40 ± 32.19	0.786

Data are shown as mean±SD or *n* (%). *CP*, cerebral palsy; *EDACS*, Eating and Drinking Ability Classification System; *GMFCS*, Gross Motor Function Classification System.

### Nutritional status

Significant improvements were obtained in WHZ, WAZ, BAZ, weight, and height in the two groups after 6-month treatment (*p* < 0.05, Table 2). As for the changes of weight and WAZ, there were significant differences between the two groups (*p* < 0.05, Table 3). Although the HAZ *z*-scores of most participants increased, there was no significant within-group and between-group comparison difference (*p* > 0.05, Tables 2, 3).

**Table 2:** Within-group comparison of nutritional parameters and GMFM scores.

	**Baseline**	**3 months**	**6 months**	***p-*value**
**Weight (kg)**				
Formula group	11.16 ± 3.06	12.09 ± 3.28*	12.77 ± 3.20*	**<0.001**
Control group	11.28 ± 2.71	11.95 ± 2.72*	12.46 ± 2.63*	**<0.001**
Height (cm)				
Formula group	87.98 ± 12.54	89.41 ± 16.36	92.78 ± 11.93*	**0.004**
Control group	88.29 ± 11.38	90.89 ± 11.45*	92.69 ± 11.53*	**<0.001**
**weight-for-height z-score**				
Formula group	−1.35 ± 0.70	−1.09 ± 0.81*	−0.79 ± 0.88*	**<0.001**
Control group	−1.35 ± 1.05	−1.19 ± 0.95*	−1.05 ± 1.14*	**0.048**
**weight-for-age z-score**				
Formula group	−1.82 ± 0.77	−1.57 ± 0.73*	−1.43 ± 0.78*	**<0.001**
Control group	−1.92 ± 0.96	−1.77 ± 0.94*	−1.71 ± 0.95*	**0.004**
**height-for-age z-score**				
Formula group	−1.61 ± 1.16	−1.48 ± 1.04	−1.52 ± 1.02	0.258
Control group	−1.78 ± 1.33	−1.67 ± 1.30	−1.70 ± 1.33	0.443
**BMI-for-age z-score**				
Formula group	−1.20 ± 0.74	−0.93 ± 0.86^†^	−0.67 ± 0.89*	**<0.001**
Control group	−1.19 ± 1.09	−1.05 ± 1.06	−0.97 ± 1.29	0.157
**Gross motor function measure**				
Formula group	53.76 ± 32.08	59.16 ± 30.77*	63.40 ± 30.47*	**<0.001**
Control group	54.40 ± 32.19	57.99 ± 31.44*	59.98 ± 31.45*	**<0.001**

Values are presented as mean ± SD. Analyzed by one-way repeated measures analysis of variance (bold values represent *p* < 0.05).

* Significantly different than baseline, *p* < 0.05.

† Significantly different than baseline, *p* < 0.01.

**Table 3:** Between-group comparison of changes in nutritional parameters and GMFM scores.

**Nutritional parameters**	**Changes**	**Formula group** **(*n* = 54)**	**Control group** **(*n* = 56)**	***p***
Weight (kg)	Δ(Visit 1–Baseline)	0.80 (0.58–1.20)	0.65 (0.43–1.00)	**0.039**
	Δ(Visit 2–Baseline)	1.60 (1.08–2.00)	1.10 (0.83–1.48)	**<0.001**
Height (cm)	Δ(Visit 1–Baseline)	3.00 (1.45–4.00)	2.15 (1.00–3.78)	0.365
	Δ(Visit 2–Baseline)	5.00 (3.00–6.28)	3.75 (2.40–5.98)	0.209
Weight-for-height z-score	Δ(Visit 1–Baseline)	0.19 (−0.17–0.64)	0.19 (−0.25–0.65)	0.864
	Δ(Visit 2–Baseline)	0.55 (0.02–1.06)	0.30 (−0.05–0.93)	0.315
Weight-for-age z-score	Δ(Visit 1–Baseline)	0.20 (−0.02–0.50)	0.16 (−0.04–0.39)	0.367
	Δ(Visit 2–Baseline)	0.35 (0.06–0.74)	0.16 (−0.17–0.42)	**0.024**
Height-for-age z-score	Δ(Visit 1–Baseline)	0.13 (−0.22–0.49)	0.05 (−0.36–0.53)	0.588
	Δ(Visit 2–Baseline)	0.15 (−0.31–0.50)	0.00 (−0.47–0.43)	0.650
Body mass index-for-age z-score	Δ(Visit 1–Baseline)	0.22 (−0.19–0.70)	0.15 (−0.35–0.65)	0.411
	Δ(Visit 2–Baseline)	0.40 (−0.06–0.94)	0.18 (−0.31–0.91)	0.093
Gross Motor Function Measure	Δ(Visit 1–Baseline)	3.02 (1.06–6.17)	1.48 (0.48–4.06)	**0.021**
	Δ(Visit 2–Baseline)	6.29 (4.28–12.97)	2.92 (0.91–6.16)	**<0.001**

Values are presented as median. IQR, interquartile range.

Analyzed by the Mann–Whitney *U*-test (bold values represent *p* < 0.05).

**Figure 2:**
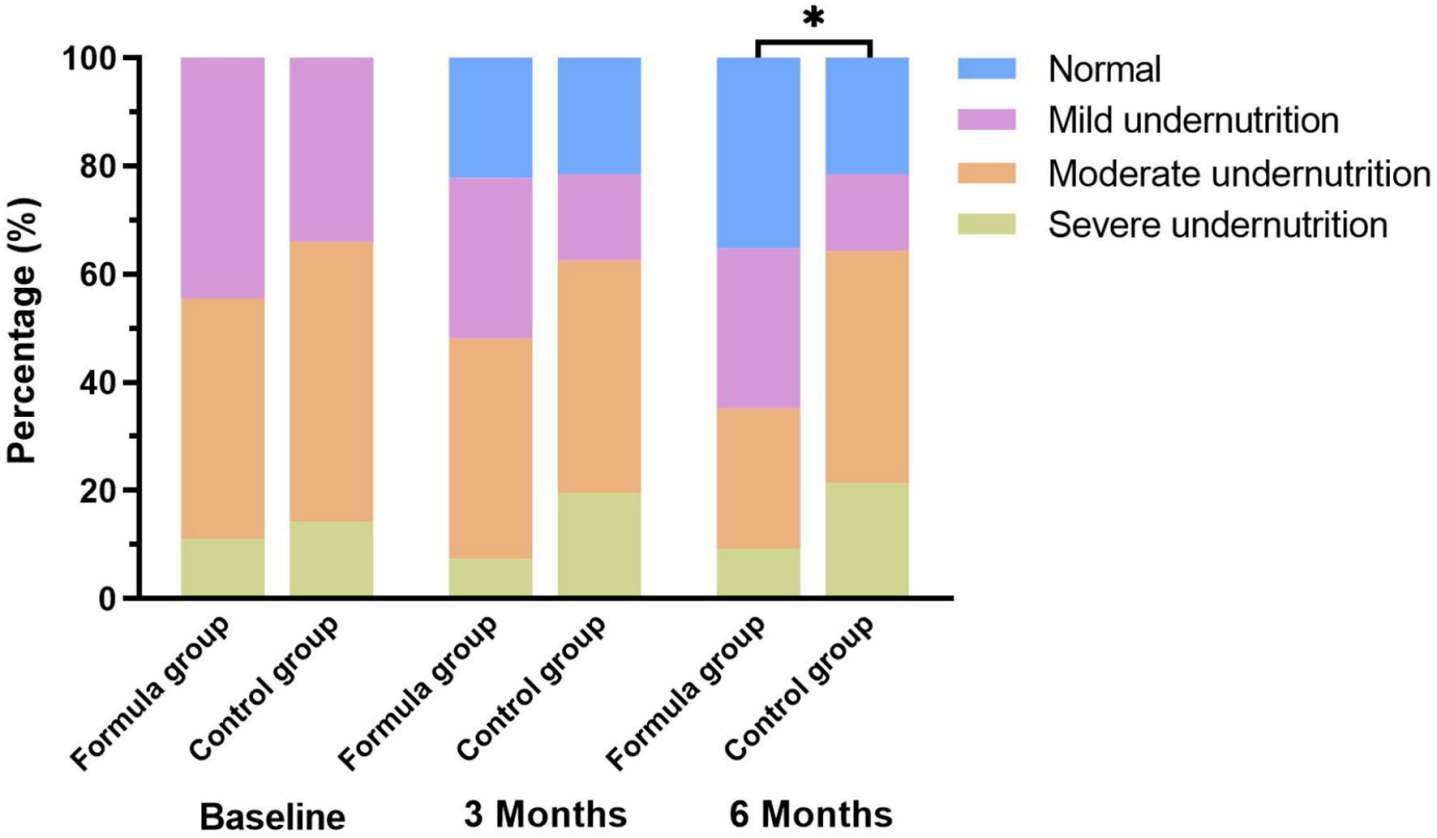
Changes of nutritional classification among the study groups. *p < 0.05: significantly different in nutritional classifications between the two groups.

**Figure 3:**
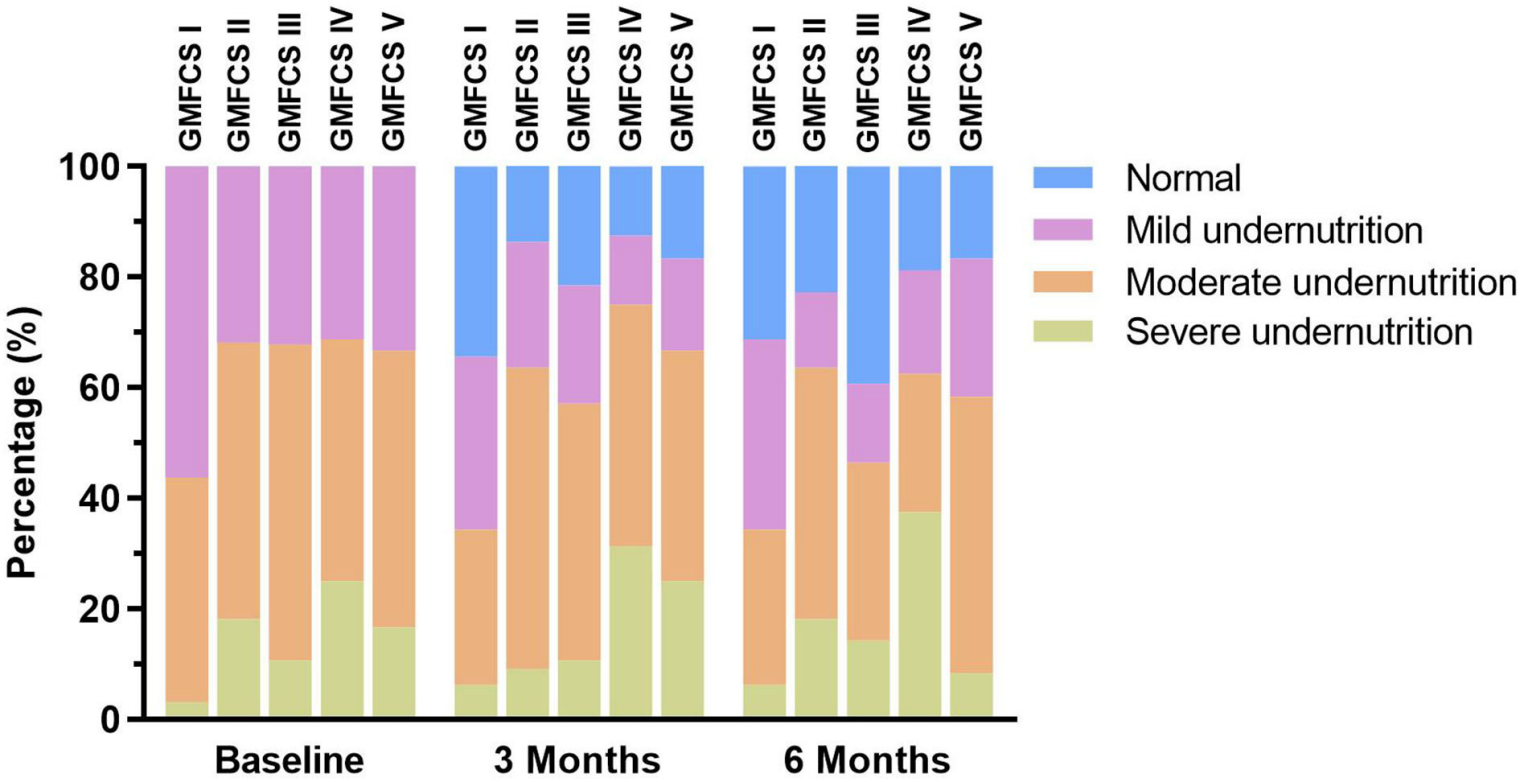
Changes in nutritional status before and after nutritional intervention at each Gross Motor Function Classification System (GMFCS) level.

What's more, we found that the rate of normal nutritional status that was improved from undernutrition in the formula group was significantly higher than that in the control group (35.2 vs. 21.4%, *p* < 0.05). The changes in nutritional status of the two study groups at different time points are illustrated in Figure 2. The changes in nutritional status before and after nutritional intervention at each GMFCS level are shown in Figure 3.

### Gross motor function

The GMFM scores of the two groups were significantly improved after 3- and 6-month intervention (*p* < 0.05, Table 2). Furthermore, the increments of GMFM scores in the formula group were significantly higher than that of the control group at both 3 and 6 months after intervention (*p* < 0.05, Table 3).

### Adverse events

**Figure 4:**
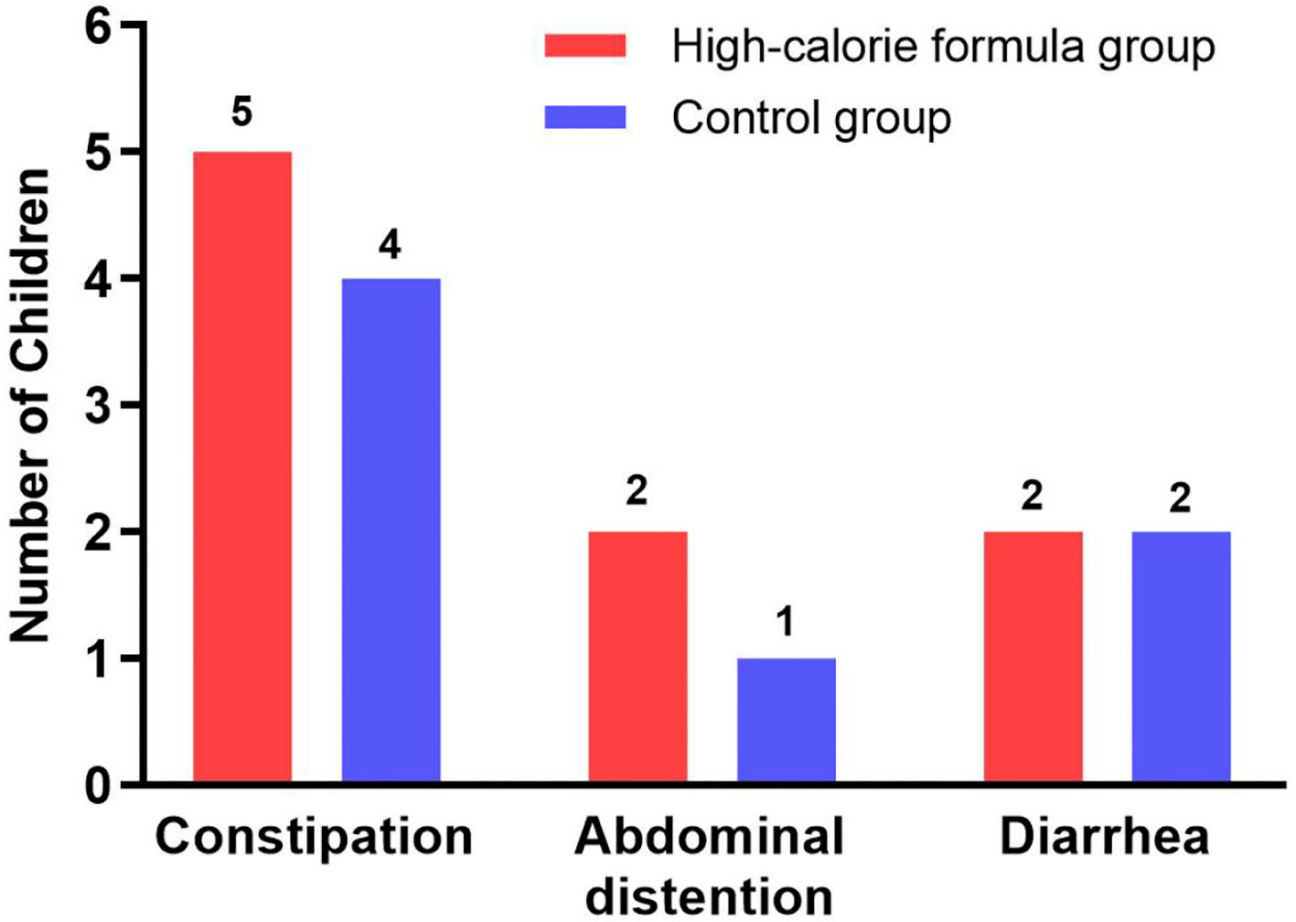
Gastrointestinal tolerance parameters of the formula and control groups during the study period.

During the intervention period, gastrointestinal symptoms occurred in 9 participants (16.7%) of the formula group resulting from signs of enteral feeding intolerance, of which five participants had constipation, two participants had abdominal distension, and two participants had diarrhea. In the control group, 7 children (12.5%) experienced gastrointestinal intolerance, of which four children experienced constipation, one child experienced abdominal distension, and two children experienced diarrhea. No serious adverse events in the two groups were noted, and the incidence rate of gastrointestinal symptoms did not differ significantly between the two groups (*p* > 0.05), namely, both feeds were equally well tolerated. This information is summarized in Figure 4.

## Discussion

This might be the first randomized controlled trial to compare the efficacy and tolerance of oral nutritional supplements between high-calorie formula (1.0 kcal/ml) and Chinese diet food in undernourished children with CP. The present study suggested that the increase of weight and WAZ in the high-calorie formula protocol was significantly higher than that in the Chinese diet protocol after 6 months intervention, which indicated the high-calorie formula may help promote weight gain and grow better in children with CP. What's more, our findings demonstrated that nutritional intervention using the high-calorie formula would also contribute to improving the gross motor function of children with CP.

Although prescribed energy intake was homologous for both groups in this study, there were significantly higher gains of both body weight and gross motor function in the formula group. Oral nutritional supplement using a high-calorie formula during a six-month intensive nutritional supplementation period produced distinct changes in anthropometric and gross motor function parameters in children with CP and mild to severe undernutrition. Previous studies evaluating the effect of enteral feeding on anthropometric parameters have shown similar findings. For example, Arrowsmith F et al. found that undernourished children with quadriplegic CP showed significant improvements in body fat and protein by means of gastrostomy tube feeding (25), while another study found that the standard polymeric formula (1.0 kcal/ml) *via* a gastrostomy tube represented a safe and effective nutritional intervention in children with neurologically impaired and undernutrition (26). Similarly, Soylu OB et al. used polymeric products (1.0 kcal/ml) for nutritional support in 31 children with spastic quadriplegia for 6 months, which exhibited improvements in anthropometric findings, including weight, height, weight *z*-scores, and mid-arm circumference (27). What's more, our study indicated that the effective rate of the formula group was higher than that of the Chinese diet group after a 6-month follow-up. In other words, the nutritional category of more participants in the formula group was improved, which elucidated that nutritional intervention with a high-calorie formula was more effective than with Chinese diet food.

The better efficacy of enteral formula may be explained in different aspects. It is possible that the actual food intake in the Chinese diet food group was less than what we had recommended according to calculations of the children's daily needs. The preparation of home-made food in the quantity and composition corresponding to accurate instructions of a clinical dietitian is a challenging task for parents, compared with the provision of the recommended daily volume of enteral formulas. Parents might thus gradually start to improvise, both in the selection of foods and in the amounts. Even with the same volume of feeding, the daily energy intake may alter owing to a change in the selection of foods and/or their amount. Additionally, the better efficacy of high-calorie enteral formula might be that with the same calorie energy, the volume of the high-calorie formula is smaller, which might contribute to alleviating gastric retention and gastroesophageal reflux. It was reported that slow gastric emptying and gastroesophageal reflux were common in children with CP (28, 29). These children tend to tolerate poorly large volumes of food. Chinese diet food is characterized by complicated cooking methods, rich sauces, and various types of food. In the Chinese context and culture, parents are used to feeding their children rice porridge, a kind of soft food made by boiling meals of grains or legumes in milk or water until thick, such as chopped meat porridge, vegetable porridge, and egg porridge. In addition, Chinese caregivers like to feed the children with soup, a kind of liquid food often containing pieces of solid food with meat, fish, or vegetable stock, especially in the coastal areas of South China. However, these semi-solid foods generally contain lower energy density and less nutrients. As enteral formula (1.0 kcal/ml) has a higher average energy density than daily food, the volumes of daily food needed to cover the same energy and nutrient intake were higher, compared with the high-calorie formula. Although vomiting or regurgitation was not reported, the volumes of food prescribed in the intervention were more than the ones they were used to before the study for most of the participants. On the other hand, there is also a probability that the biological availability and utilization of nutrients of high-calorie formula was significantly better than in Chinese diet food. Studies involving undernourished children in developing countries and animal-model studies showed that gastrointestinal structure and function were affected in the circumstances of severe undernutrition (30–33). Therefore, the digestive and absorptive functions of some nutrients in the subjects may be impaired, and the high-calorie formula with special medical use could just help improve gastrointestinal digestion and absorption.

Interestingly, we found the height of participants grew slowly and the height-related nutritional parameters (i.e., H/A *z*-scores) of the two groups did not change significantly after 3 or 6 months of nutritional rehabilitation. It seems probable that the long bones (such as tibia, fibula, and femur) of children with CP have a tendency to lack stress stimulation, because of the spastic limbs, limited functional mobility, and lack of weight bearing, which may have a negative impact on height growth and may result in osteoporosis (34, 35). This suggests that the bone health of children in CP should be well recognized.

Motor dysfunction is the core symptom of children with CP and several studies have demonstrated that there are numerous techniques to promote motor function (36). However, there is little research to explore the effect of nutritional intervention on motor function of children with CP. Our study revealed that the gross motor function of the two nutritional protocols was significantly improved after 3 and 6 months of intervention, and the increase in the high-calorie formula group was higher than that in the Chinese diet group. It is known that protein is the basic component of muscle structure, and energy is the foundation of physical activity. During the stable and recovery phase of the disease progression, the catabolism will be converted into anabolism and nutritional support should emphasize on increasing protein and energy intake to promote recovery as well as catch-up growth. A high-calorie formula contains higher energy density and protein content, which might help facilitate the muscle growth and energy intake of children. Moreover, only children with sufficient energy supply can carry out physical activities and rehabilitation exercises on a daily basis. Accordingly, the motor function of children who received a high-calorie formula for nutritional intervention might be greatly improved.

In the present study, we used an exercise training protocol with the intensity of 3 sets of 8–12 repetitions of 50–85% repetition maximum and the duration of 30 min per session that was recommended for children with CP at different GMFCS levels (18). Controlling the training intensity was essential for this randomized controlled study to avoid the influence of confounding factors. Thus, participants at different GMFCS levels in the two groups were given a similar intensity of training, and it was believed that the impact of intensity of the training on function between the groups was basically consistent. Furthermore, the subjects had been undergoing rehabilitation training before they were recruited in this study. That is to say, the subjects have been undergoing rehabilitation training regardless of whether there is a nutritional intervention. Hence, the difference of impact of training intensity on energy requirements between groups was negligible. Apart from this, subjects were regularly followed up every 2–4 weeks to monitor the changes in energy demands and adjust the recommended intake at any time if necessary.

Tolerance is an important aspect of adverse events in oral nutritional support. In our study, 16.7% of children in the formula group had gastrointestinal intolerance, such as constipation, abdominal distention, and diarrhea, while 12.5% of children in the control group developed gastrointestinal intolerance. There were no significant differences between the groups with regard to the incidence rate of gastrointestinal symptoms, suggesting that the high-calorie formula was well tolerated among children with CP. It is in accordance with the earlier studies (37, 38).

The relatively short-term follow-up and disregard of the development of metabolic syndrome were limitations, which should be considered in light of the fact that abnormal metabolism may occur amid nutritional intervention in the long term. In addition, we did not collect nutritional data from other dimensions, such as body composition and laboratory biochemical indicators, because of limited conditions. Finally, our study did not include children who were fed *via* a tube, therefore the results may not be generalized to the whole population of children with CP. Despite the aforementioned limitations, the results of this randomized controlled trial suggest that compared with the high-calorie enteral formula, even a professionally planned diet using Chinese diet food with parents or caregivers is less effective in terms of weight gain and motor function development of undernourished children with CP.

In conclusion, nutritional intervention with a high-calorie formula may effectively improve weight gain and gross motor function of children with CP for up to 6 months without serious adverse events. Future research using a more rigorous study design and a larger sample size to compare effectiveness of both feeding regimes is warranted.

## Data availability statement

The raw data supporting the conclusions of this article will be made available by the authors, without undue reservation.

## Ethics statement

The studies involving human participants were reviewed and approved by the Medical Research Ethics Committee of Guangzhou Women and Children's Medical Center, Approval No: 202023301. Written informed consent to participate in this study was provided by the participants' legal guardian/next of kin.

## Author contributions

KX conceived and designed this study, carried out project management, and provided research funding. YZ, LH, TP, and LL conducted data collection and manuscript writing. HZ charted the figures and tables. YX and XY performed data statistical analysis. YH and ZC carried out the literature search. YX and JL committed to participant recruitment. KX, HT, and XH revised and reviewed the manuscript. All authors have reviewed and approved the content of this manuscript.

## Funding

The study was supported by the Natural Science Foundation of Guangdong Province (2021A1515011303, 2021A1515012543, and 2019A1515010420), and grants from the Science and Technology Program of Guangzhou (202102010205) and the Featured Clinical Technique of Guangzhou (2019TS55).

## Conflict of interest

The authors declare that the research was conducted in the absence of any commercial or financial relationships that could be construed as a potential conflict of interest.

## Publisher's note

All claims expressed in this article are solely those of the authors and do not necessarily represent those of their affiliated organizations, or those of the publisher, the editors and the reviewers. Any product that may be evaluated in this article, or claim that may be made by its manufacturer, is not guaranteed or endorsed by the publisher.
